# Clinical characteristics and treatment strategies of ovarian teratomas associated with anti-N-methyl-D-aspartate receptor encephalitis based on a case series

**DOI:** 10.3389/fneur.2026.1817027

**Published:** 2026-10-29

**Authors:** Heqin Yang, Chun Fang, Shijun Wang

**Affiliations:** Department of Obstetrics and Gynecology, Xuanwu Hospital, Capital Medical University, Beijing, China

**Keywords:** anti-NMDAR encephalitis, disease, ovarian teratoma, prognosis, retrospective study

## Abstract

**Introduction:**

Anti-N-methyl-D-aspartate receptor (anti-NMDAR) encephalitis is a potentially life-threatening yet reversible complication associated with ovarian teratoma. This study aims to systematically analyze the clinical features of anti-NMDAR encephalitis related to ovarian teratoma, with the goal of enhancing our understanding of the disease.

**Methods:**

A retrospective analysis was performed on 17 patients with anti-NMDAR encephalitis associated with ovarian teratomas, who were admitted to Xuanwu Hospital, Capital Medical University, from August 2015 to August 2025. A systematic analysis was performed on their complete clinical data, including demographic characteristics, prodromal symptoms, typical neurological and psychiatric manifestations, cerebrospinal fluid and neuroimaging findings, anti-NMDAR antibody in cerebrospinal fluid, pathological features of the ovarian teratomas, comprehensive treatment strategies and follow-up outcomes.

**Results:**

In this study, the age of the included patients was 25 years (21, 29). The time from symptom onset to diagnosis was 16 days (10.5, 27). The time from hospital admission to diagnosis was 4 days (2.5, 7). Two patients had a history of teratomas and methotrexate treatment. 15 patients (15/17, 88%) were directly admitted to the intensive care unit, while only 2 (2/17, 12%) were treated in general wards. The patients predominantly presented with multiple neurological abnormalities without clinical signs of teratoma compression. All patients received immunotherapy and tumor resection, and all patients achieved clinical improvement. At 3 months after surgery, mRS ≤ 2 was achieved in patients (4/17, 24%), mRS 3–4 in 5 paitents (5/17, 29%), and mRS 5 in 8 patients (8/17, 47%). Although all patients met the pre-defined criterion of clinical improvement in at least one neurological symptom, functional recovery remained limited.

**Conclusion:**

A history of teratoma and methotrexate treatment may be associated with a higher risk of teratoma-associated anti-NMDAR encephalitis; however, this observation was based on only two patients and warrants further investigation. The disease is characterized by acute onset and rapid progression, and clinical improvement can be achieved through multidisciplinary collaboration combined with prompt immunotherapy and tumor resection.

## 1 Introduction

Anti-N-methyl-D-aspartate receptor (anti-NMDAR) encephalitis is an inflammatory disorder of the central nervous system characterized by IgG antibodies directed against the NR1 subunit of the NMDA receptor (NMDAR) (1–4). It can produce a constellation of symptoms resembling infectious encephalitis, and some patients may present exclusively with neurological and psychiatric manifestations (5). Among patients diagnosed with anti-NMDAR encephalitis (NMDAR-E), an underlying neoplasm is identified in approximately 25–40% of cases, and around 90% of the associated tumors are ovarian teratomas (6). More than 80% of patients with anti-NMDAR encephalitis achieve recovery (7); however, a small proportion may be left with sequelae such as neurological dysfunction, with an associated mortality rate of approximately 7% (8). Several studies have characterized anti-NMDAR encephalitis in the setting of ovarian teratoma (9, 10). He et al. found that brain MRI and electroencephalography lacked specificity for the diagnosis of the disease, whereas detection of anti-NMDAR antibodies in cerebrospinal fluid was crucial for diagnosis (10).

Chefdeville et al. analyzed the immunopathological features of 27 ovarian teratomas associated with anti-NMDAR encephalitis. Their results showed that the neuroglial tissue of three NMDAR-E-associated mature teratomas exhibited histopathological characteristics of central nervous system neuroglial tumors. NMDAR-E-associated teratomas also differed from sporadic ovarian teratomas by consistent and prominent infiltration of the nervous tissue component by immune cells (6). These findings provide important experience for diagnosing and treating the disease. However, because of its rarity, most studies focus on single cases, and systematic analyses remain lacking. In addition, patients often initially present with psychiatric or neurological symptoms and are referred to psychiatric or neurological departments, so the interval from tumor detection to definitive surgical intervention tends to be prolonged even when a teratoma is present. This delay may cause patients to miss the optimal window for treatment. Therefore, there is an urgent need to analyze the clinical characteristics and treatment strategies, which could improve patient prognosis.

In this study, 17 cases of ovarian teratomas associated with anti-NMDAR encephalitis were retrieved and analyzed to improve clinicians’ understanding of the disease and facilitate early diagnosis and treatment.

## 2 Materials and methods

### 2.1 Patient samples

This retrospective study was based on clinical data from 17 patients diagnosed with anti-NMDAR encephalitis coexisting with ovarian teratoma, all of whom were admitted to Xuanwu Hospital, Capital Medical University between August 2015 and August 2025. Clinicopathologic characteristics were systematically retrieved from archived medical records. The patients met the diagnostic criteria of anti-NMDAR encephalitis (5, 11) and ovarian teratomas. The inclusion criteria were as follows: (1) positive anti-NMDAR antibody in cerebrospinal fluid; (2) rapid onset (within 3 months) with one or more of the following: abnormal behavior or mental state; cognitive impairment; speech disorders; epileptic seizure; involuntary movement or movement disorders; decreased level of consciousness; central ventilation disorder or autonomic nervous dysfunction; (3) ovarian teratoma was confirmed by postoperative pathology. The exclusion criteria were as follows: (1) exclusion of other causes of encephalitis; (2) lack of clinicopathologic data. All anti-NMDAR antibody tests were performed using cell-based immunofluorescence assays (CBA).

### 2.2 Data collection

We collected demographic information, laboratory test results, ultrasound and imaging findings, and postoperative pathological data from the included patients. Among these findings, abnormal cranial magnetic resonance imaging (MRI) results were defined as the presence of specific parenchymal-related abnormalities, including medial temporal lobe or hippocampal involvement (e.g., FLAIR hyperintensity or atrophy), cortical or subcortical FLAIR hyperintensities, leptomeningeal enhancement, white-matter lesions, and ventriculomegaly. Isolated age-related changes were not classified as abnormal. Abnormal electroencephalography (EEG) results were defined as any reported abnormality, including diffuse or multifocal slow-wave activity, delta waves or extreme delta brushes, focal slowing, epileptiform discharges, and electrographic seizures. Cerebrospinal fluid (CSF) opening pressure, white blood cell count, and protein levels were interpreted based on institutional reference ranges (opening pressure: 80–180 mmH₂O; white blood cell count: 0–5 × 10^6^/L; protein: 15–45 mg/dL). Detailed findings for each patient are presented in Table 1. The modified Rankin Scale (mRS) was employed to comprehensively evaluate patients’ neurological status at admission and throughout postoperative follow-up. As a widely recognized instrument, the mRS assesses functional independence and the degree of disability in individuals recovering from stroke or other neurological impairments (12). In our study, clinical improvement was defined as documented resolution or notable reduction in at least one of the major neurological symptoms listed in the diagnostic criteria. Besides, an mRS score ≤2 indicated a favorable prognosis, a score of 3–4 signified moderate neurological disability, and a score of 5 represented severe neurological disability.

**Table 1:** Examinations of the patients.

Case No	Pelvic imaging (US)	Brain MRI	EEG	Specific oligoclonal band in CSF	NMDAR antibody in CSF at admission
1	Teratoma	Multiple punctate abnormal signal foci in the bilateral frontoparietal cerebral white matter	Extensively highly abnormal	Positive	1:100
2	Mixed solid-cystic mass	Bilateral frontal and temporal gyri appear plump with subtle hyperintensity on FLAIR	Generalized diffuse slow waves	Positive	1:100
3	No abnormality	No abnormalities	Moderately abnormal. Scattered 3 Hz slow waves occasionally observed in bilateral frontal poles and frontal leads, with superimposed 25 Hz fast waves (*δ* brushes)	Positive	1:100
4	Pelvic mixed solid-cystic	No abnormalities	No abnormalities	Negative	1:32
5	Cyst	Slightly hyperintense signals in the mesial temporal lobes, insulae, and hippocampi	Mildly abnormal, with a moderately increased amount of medium-amplitude slow waves intermixed in the background	Positive	1:100
6	Teratoma	Suspiciously increased signal in the left hippocampus on FLAIR	No definite epileptiform discharges	Negative	1:320
7	Teratoma	Extensive linear enhancement along the sulci of bilateral cerebral hemispheres	No abnormalities	Negative	1:32
8	First: Right ovarian cyst./Teratoma Second: Teratoma.	Bilateral temporal horns, lateral ventricles, and cerebral sulci are widened; signal intensity in the right hippocampus and mesial temporal lobe is slightly higher than on the left; left hippocampal atrophy	Diffuse chronic *δ* activity with visible *δ* brushes	Positive	1:320
9	Teratoma	No abnormalities	Slow waves with no epileptiform discharges	Positive	1:100
10	Teratoma	Mild atrophic changes of the brain; inhomogeneous signal within the pituitary gland	Generalized medium- to high-amplitude slow waves, status epilepticus	Positive	1:32
11	Teratoma	No abnormalities	No abnormalities	Negative	1:320
12	Teratoma	Punctate abnormal signal foci adjacent to the left lateral ventricle posterior horn and in the right frontal subcortical area	Moderately abnormal	Negative	1:1,000
13	–	No abnormalities	No abnormalities	Negative	1:320
14	Teratoma	Supratentorial ventriculomegaly with periventricular abnormal signal changes	Scattered medium- to high-amplitude 2–3 Hz complex slow waves and slow activity in the bilateral frontal regions. Scattered low- to medium-amplitude low-voltage 19–26 Hz beta rhythms and *β* activity in all leads	Negative	1:320
15	First: Teratoma Second: Hyperechoic lesion in left ovary.	No abnormalities	Rhythmic delta (*δ*) waves	Positive	Strongly positive
16	Teratoma	Abnormal signal in the splenium of the corpus callosum with restricted diffusion on DWI	Background predominantly composed of beta waves; no epileptiform discharges	Positive	1:32
17	Mixed solid-cystic mass in right adnexa	No abnormalities	Extensive abundant low- to medium-amplitude slow waves with numerous low-amplitude fast waves continuously discharging (suspicious for *δ* brushes)	Positive	1:100

US, Ultrasound.

### 2.3 Statistical analysis

All statistical analyses were performed using SPSS 29.0. Quantitative data are expressed as median with interquartile range, while categorical data are presented as number (%).

## 3 Results

### 3.1 General demographic characteristics of 17 patients

The basic clinical information of the 17 patients is shown in Table 2. All patients were young females, with ages ranging from 16 to 40 years (median, 25 years; IQR, 21–29 years), including two patients under 18 years of age. Six patients had a history of delivery. The time from symptom onset to diagnosis ranged from 5 to 34 days (median, 16 days; IQR, 10.5–27 days), and the time from hospital admission to diagnosis ranged from 1 to 25 days (median, 4 days; IQR, 2.5–7 days). Review of the medical history revealed that one patient (Case 5) had undergone right ovarian teratoma excision 5 years before disease onset and had surgery for ectopic pregnancy 3 years prior to onset. One patient (Case 6) was found to have a left ovarian teratoma on pelvic ultrasound 1 month before disease onset. One patient (Case 8) had undergone surgery for ectopic pregnancy 1 year before disease onset. Another patient (Case 16) gave birth to twins, one of whom was diagnosed with spinal muscular atrophy, while the other was a gene carrier. Both patients with a prior history of teratoma (Cases 5 and 6) self-reported previous methotrexate treatment for ectopic pregnancy; however, detailed regimens, doses, and treatment timelines were not retrievable from the medical records.

**Table 2:** The general information and clinical characteristics of the patients.

Case	Age (years)	Days from onset to diagnosis	Days from admission to diagnosis	Mode of onset	Prodromal symptoms	Initial symptoms	Typical symptoms
1	31	33	15	Acute	Fever, Headache (Sudden-onset; Persistent)	Cognitive Impairment	Psychiatric/behavioral abnormalities, Seizures, Involuntary movements, Loss of consciousness, Cognitive impairment.
2	23	5	2	Subacute	None	Psychiatric/behavioral abnormalities	Psychiatric/behavioral abnormalities, Seizures, Involuntary movements, Loss of consciousness, Central hypoventilation.
3	27	23	1	Acute	Fever, Headache (Sudden-onset; Holocranial; Distending pain; Persistent)	Diplopia (Double vision)	Psychiatric/behavioral abnormalities, Cognitive impairment, Loss of consciousness.
4	25	10	1	Acute	None	Diplopia (Double vision)	Psychiatric/behavioral abnormalities, Involuntary movements, Loss of consciousness.
5	28	10	3	Subacute	Headache (Sudden-onset; Occipital and frontal region; Dull pain; Lasting 3–5 min with spontaneous resolution; Occurring several times daily)	Slowed response	Decreased level of consciousness, Cognitive impairment.
6	30	16	5	Acute	Headache (Holocranial; Paroxysmal; Dull pain)	Psychiatric/behavioral abnormalities	Psychiatric/behavioral abnormalities, Seizures, Involuntary movements, Loss of consciousness.
7	26	16	4	Acute	Fever	Psychiatric abnormalities	Psychiatric/behavioral abnormalities, Seizures, Involuntary movements, Cognitive impairment.
8	27	11	3	Acute	Fever, Headache (Insidious onset; spontaneous resolution; Worsened 3 days later)	Psychiatric abnormalities	Psychiatric/behavioral abnormalities, Seizures, Involuntary movements, Cognitive impairment, Loss of consciousness.
9	18	23	4	Acute	Fever, Headache (Sudden-onset; Persistent)	Psychiatric/behavioral abnormalities	Psychiatric/behavioral abnormalities, Seizures, Involuntary movements, Loss of consciousness, Central hypoventilation.
10	16	7	3	Acute	None	Psychiatric/behavioral abnormalities	Psychiatric/behavioral abnormalities, Seizures, Involuntary movements, Loss of consciousness, Central hypoventilation.
11	24	14	5	Acute	Fever, Headache (Sudden-onset; Persistent)	Psychiatric/behavioral abnormalities	Psychiatric/behavioral abnormalities, Involuntary movements, Cognitive impairment, Motor dysfunction.
12	32	25	4	Acute	Fever, Headache (Sudden-onset; Occipital region; Dull pain; Persistent)	Psychiatric abnormalities	Psychiatric/behavioral abnormalities, Seizures, Involuntary movements, Loss of consciousness, Central hypoventilation.
13	40	29	2	Acute	Headache (Sudden-onset; Bilateral temporal; Stabbing; Paroxysmal)	Memory decline, Personality changes	Psychiatric/behavioral abnormalities, Seizures, Involuntary movements.
14	19	34	25	Acute	None	Psychiatric/behavioral abnormalities	Psychiatric/behavioral abnormalities, Seizures, Involuntary movements, Cognitive impairment, Loss of consciousness, Central hypoventilation.
15	24	15	9	Subacute	Fever, Headache (Sudden-onset; Bilateral parietal; Stabbing pain)	Speech disorder	Psychiatric/behavioral abnormalities, Seizures, Involuntary movements, Cognitive impairment, Motor dysfunction, Loss of consciousness, Central hypoventilation.
16	24	30	1	Subacute	None	Psychiatric/behavioral abnormalities	Psychiatric/behavioral abnormalities, Seizures, Involuntary movements, Motor dysfunction, Loss of consciousness, Central hypoventilation.
17	17	21	4	Acute	Fever	Psychiatric/behavioral abnormalities	Psychiatric/behavioral abnormalities, Seizures, Involuntary movements, Motor dysfunction, Loss of consciousness, Central hypoventilation.

### 3.2 Clinical characteristics

Among the 17 patients, 13 (13/17, 76%) had an acute onset and 4 (4/17, 24%) had a subacute onset. Prodromal symptoms, including headache, fever, or both, were present in 12 patients (12/17, 71%), while the remaining 5 patients had no prodromal symptoms. The initial symptoms mainly included psychiatric or behavioral abnormalities, cognitive impairment, and diplopia. Specifically, 12 patients (12/17, 71%) presented with psychiatric or behavioral abnormalities as the initial symptom, 3 patients (3/17, 18%) presented with cognitive impairment as the first symptom, and 2 patients (2/17, 12%) presented with diplopia as the first symptom. The most common clinical manifestations were psychiatric or behavioral abnormalities (16/17, 94%) and involuntary movements (15/17, 88%), followed by seizures (13/17, 76%) and impaired consciousness (13/17, 76%), cognitive impairment (8/17, 47%) and central hypoventilation (8/17, 47%). None of the patients exhibited clinical signs related to teratoma compression, such as abdominal pain or distension, at the time of admission. The details are shown in Table 2.

### 3.3 Clinical examinations

Pelvic examination findings are presented in Table 1. Sixteen patients underwent pelvic evaluation via ultrasound (transvaginal ultrasound for married patients, transabdominal ultrasound for unmarried patients). Ultrasound detected ovarian teratoma in 11 patients. In 4 patients, teratoma was not identified on ultrasound but was detected on subsequent CT and/or MRI. One patient (Case 5) had imaging findings suggestive of ovarian cyst/cystadenoma on ultrasound, CT and MRI; however, the final pathological diagnosis was teratoma.

Cranial MRI findings were abnormal in 10 patients (10/17, 59%) and showed no abnormality in 7 patients (7/17, 41%). The abnormalities mainly involved the medial temporal lobes or hippocampi, cerebral white matter, leptomeninges, or the ventricular system, as detailed per patient in Table 1. Electroencephalography (EEG) showed abnormalities in 12 patients (12/17, 71%). The most common abnormality was diffuse or multifocal slow-wave activity (7/17, 41%), followed by delta waves or delta brushes (4/17, 24%); one patient exhibited status epilepticus on EEG (1/17, 6%). The specific EEG findings for each patient are detailed in Table 1.

Cerebrospinal fluid (CSF) detection showed positive anti-NMDA receptor antibodies and elevated white blood cell counts in all 17 patients. All pressure readings were obtained in the lateral decubitus position. CSF-specific oligoclonal bands were positive in 10 patients (Table 1). Elevated CSF pressure was observed in 10 patients, and increased CSF protein was found in 4 patients. The median CSF pressure was 200 mmH₂O (IQR: 150, 245), the median CSF white blood cell count was 32 × 10^6^/L (IQR: 17.5, 66.5), and the median CSF protein level was 31 mg/dL (IQR: 20.1, 45.5) (Supplementary Table 1; Figure 1).

**Figure 1:**
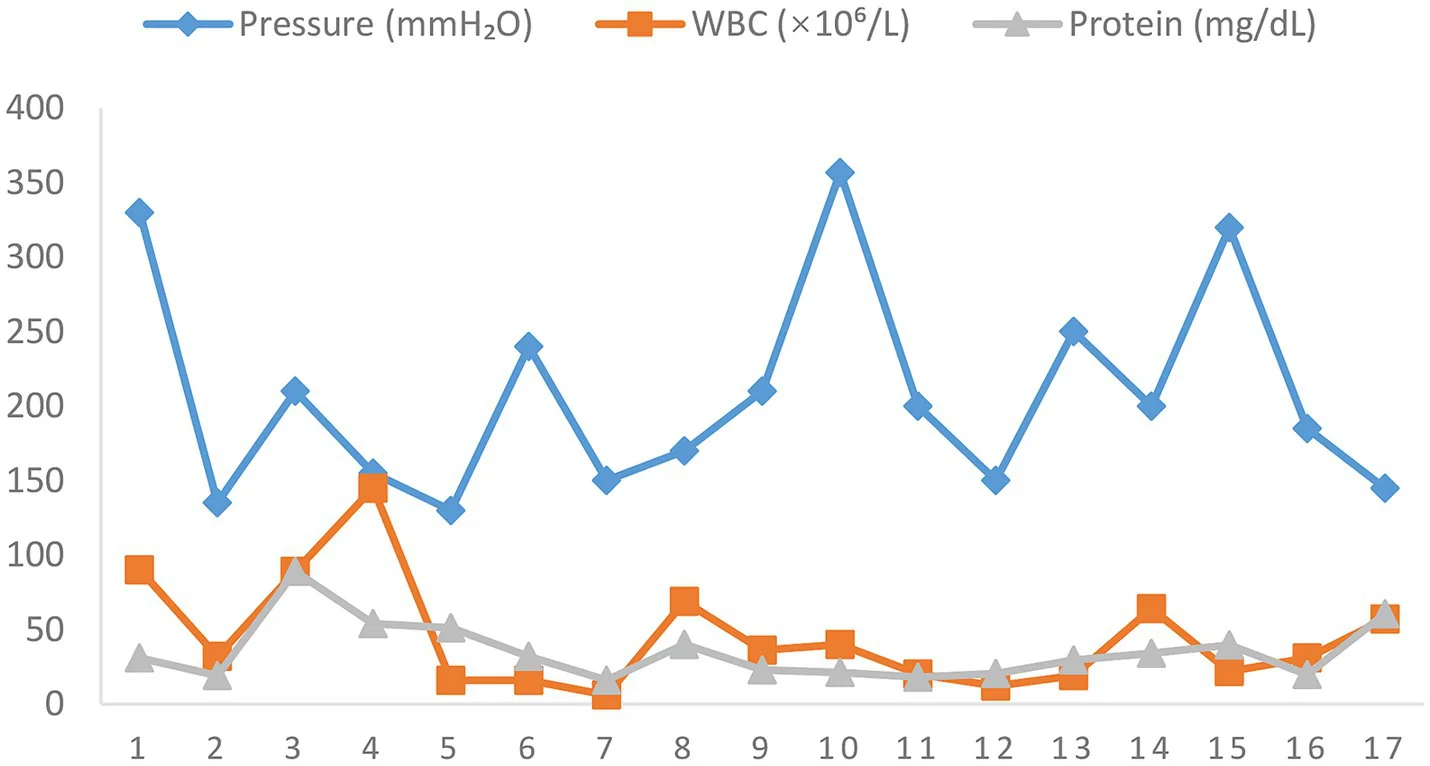
The change of pressure, WBC and protein in CSF. WBC, white blood cell; CSF, Cerebrospinal fluid.

Serological tests revealed elevated CA125 levels in 4 of the 17 patients, while CA125 was normal in the remaining patients.

### 3.4 Treatment and prognosis

All patients received first-line immunotherapy, including corticosteroids (17/17, 100%), intravenous immunoglobulin (16/17, 94%), and plasma exchange (14/17, 82%). Second-line immunotherapy was administered as rituximab alone in 9 patients, cyclophosphamide alone in 1 patient, and rituximab combined with cyclophosphamide in 1 patient. Long-term immunosuppression included mycophenolate mofetil (11 patients) and azathioprine (1 patient). One patient with refractory severe anti-NMDAR encephalitis also received bortezomib, and another patient with severe anti-NMDAR encephalitis complicated by super-refractory status epilepticus received additional tocilizumab. Fifteen patients (15/17, 88%) were admitted to the intensive care unit, whereas 2 (2/17, 12%) were treated in general wards. During hospitalization, 14 patients (14/17, 82%) required mechanical ventilation during hospitalization.

All procedures were completed laparoscopically with no conversions to open surgery, and no perioperative complications related to the operations were observed. Operative time ranged from 27 to 112 min, with a median of 35 min. Except for 2 cases that lasted over 1 h, all others were completed within 1 h. Fifteen patients underwent surgery during the acute phase, with a median interval from disease onset to surgery of 39 days (range 17–51 days). Two patients underwent surgery during the recovery phase, at 46 days and 143 days after onset, respectively. Among the 17 patients, 1 had bilateral ovarian teratomas and underwent bilateral ovarian teratoma resection. Ten patients had right-sided ovarian teratomas: 6 underwent right ovarian teratoma resection and 4 underwent right salpingo-oophorectomy. Four patients had left-sided ovarian teratomas: 1 underwent left ovarian teratoma resection and 3 underwent left salpingo-oophorectomy. Two patients required two surgical interventions during the acute phase: one first underwent right salpingo-oophorectomy for a right teratoma, followed by left ovarian teratoma resection for a left teratoma; the other underwent left ovarian teratoma resection twice for left-sided teratomas (Table 3). Postoperative pathology revealed immature teratoma with focal brain and neural tube tissue in 2 patients. The remaining 15 patients were diagnosed with mature cystic teratoma, among whom 10 showed brain/neural tissue on microscopic examination (Table 3).

**Table 3:** The treatment and prognosis of the patients.

Case No.	Days from onset to surgery	Side	Size (cm)	The type of teratoma pathology	Medical treatment (First-line)	Medical treatment (Second-line)	Medical treatment (Long-term)
1	44	Right	2 × 2.5	Mature	Steroids + IVIG + Plasma exchange	Rituximab	–
2	29	Right	3 × 4	Mature	Steroids + IVIG + Plasma exchange	–	Azathioprine
3	143	Right	2 × 1.5	Mature	Steroids + IVIG	–	–
4	25	Right	6 × 7	Mature	Steroids + IVIG + Plasma exchange	–	Mycophenolate mofetil
5	46	Right	5 × 7	Mature	Steroids		–
6	19	Left	5 × 4	Mature	Steroids + IVIG + Plasma exchange	–	–
7	46	Right	Two lesions: 2 × 1.5, 1 × 1.5	Mature	Steroids + IVIG	–	–
8	51	First: Right; Second: Left	First: 3 × 3; Second: 4 × 5	Mature	Steroids + IVIG + Plasma exchange	Cyclophosphamide	Mycophenolate mofetil
9	48	Right	1 × 1.5	Mature	Steroids + IVIG + Plasma exchange	Rituximab	Mycophenolate mofetil
10	17	Right	2 × 3	Mature	Steroids + IVIG + Plasma exchange	Rituximab	Mycophenolate mofetil
11	23	Bilateral	Right: 3 × 4; Left: 2 × 2	Both mature	Steroids + IVIG + Plasma exchange	Rituximab	Mycophenolate mofetil
12	34	Left	3 × 4	Mature	Steroids + IVIG + Plasma exchange	Rituximab	Mycophenolate mofetil
13	37	Left	3 × 4	Immature	Steroids + IVIG + Plasma exchange	Rituximab	Mycophenolate mofetil
14	48	Left	1.5 × 2	Mature	Steroids + IVIG + Plasma exchange	Rituximab + Cyclophosphamide	Mycophenolate mofetil
15	40	First: Left; Second: Left	First: 2 × 4; Second: 3 × 3	Both mature	Steroids + IVIG + Plasma exchange	Rituximab	Mycophenolate mofetil
16	39	Right	3 × 2	Immature	Steroids + IVIG + Plasma exchange	Rituximab;	Mycophenolate mofetil
17	36	Right	3 × 4	Mature	Steroids + IVIG + Plasma exchange	Rituximab	Mycophenolate mofetil

IVIG, intravenous immunoglobulin.

During treatment, the most common complications were bacterial pneumonia (15/17, 88%), electrolyte disturbances (11/17, 65%), anemia (11/17, 65%), and abnormal liver function (10/17, 59%). Other complications included respiratory failure (7/17, 41%), urinary tract infection (7/17, 41%), gastrointestinal bleeding (7/17, 41%), stress ulcer (2/17, 12%), pseudomembranous colitis (2/17, 12%), and deep vein thrombosis (5/17, 29%). Notably, 1 patient developed super-refractory status epilepticus, 3 had severe anti-NMDAR encephalitis, and 4 exhibited paroxysmal sympathetic hyperactivity syndrome.

We also assessed the prognosis of all patients at 1 and 3 months after surgery. At admission, the modified Rankin Scale (mRS) score was 3 in 1 patient and 5 in the other 16. After neurological treatment, the mRS score improved to 1 in 2 patients, while the remaining 15 still had an mRS score of 5. At 1 month after surgery, 3 patients (3/17, 18%) had a favorable outcome, 5 (5/17, 29%) had moderate neurological disability, and the remaining 9 (9/17, 53%) still had severe neurological disability. At 3 months after surgery, 4 patients (4/17, 24%) had a favorable outcome, 5 (5/17, 29%) had moderate neurological disability, and 8 (8/17, 47%) remained with severe neurological disability. Based on the pre-defined criteria described in Methods, all 17 patients achieved clinical improvement after completing immunotherapy and surgical treatment, including the two patients who required a second operation (Cases 8 and 15). No recurrence, death, or loss to follow-up occurred during the follow-up period (Figure 2; Supplementary Table 2).

**Figure 2:**
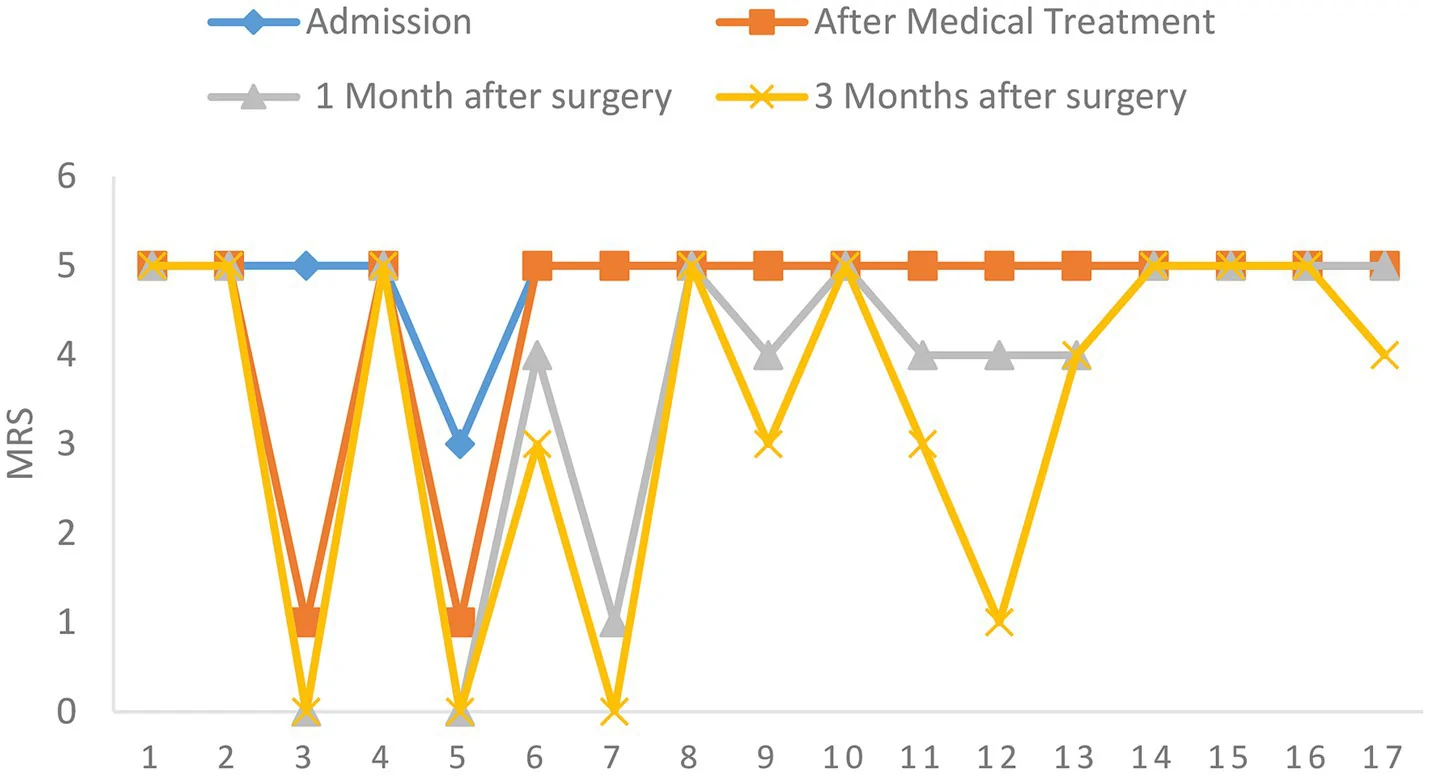
The mRS for patients.

### 3.5 Special cases

Case 5 had undergone laparoscopic right ovarian teratoma resection 5 years before disease onset. Three years before onset, laparoscopic surgery for a right tubal ectopic pregnancy revealed no abnormalities in the right ovary. After disease onset, both ultrasound and CT indicated a right ovarian cyst, while MRI suggested a right ovarian cystadenoma. At admission, the mRS score was 3. Following medical treatment, the patient showed notable improvement, with the mRS score decreasing to 1. Therefore, surgery was performed during the recovery phase, 46 days after symptom onset. Intraoperatively, a 5 × 7 cm teratoma was identified in the right ovary, and a right salpingo-oophorectomy was carried out. Pathological examination confirmed a mature ovarian teratoma, with no brain or neural tissue detected.

Case 8 had undergone left salpingectomy for ectopic pregnancy 1 year before disease onset, with no notable abnormalities observed in either ovary during that surgery. After the onset of symptoms, ultrasounds indicated an ovarian cyst or a teratoma, and MRI suggested a teratoma. Therefore, surgery was performed on day 51 after onset. Intraoperatively, a 3 × 3 cm teratoma was found in the right ovary, whereas the left ovary appeared normal. A right salpingo-oophorectomy was performed. Postoperative pathology revealed a mature cystic teratoma containing mature brain tissue. After surgery, the patient’s symptoms persisted without improvement, and the titer of anti-NMDA receptor antibodies in the cerebrospinal fluid remained 1:320 (strongly positive). Subsequent ultrasound and CT identified a teratoma in the left ovary. Therefore, a second laparoscopic surgery was performed 158 days after the first procedure. During this surgery, a 4 × 5 cm teratoma was found in the left ovary, and a left ovarian cystectomy was performed. Pathology also showed a mature cystic teratoma with abundant mature neural tissue.

Case 15 had a history of smoking and alcohol use, and high-risk sexual behavior, and her brother had a history of Japanese encephalitis and epilepsy. The first surgery was performed 40 days after disease onset. Intraoperatively, a 2 × 4 cm teratoma was found in the left ovary, and left ovarian cystectomy was performed. Pathology indicated a mature cystic teratoma without microscopic evidence of brain or neural tissue. Postoperatively, the patient showed no improvement, and anti-NMDA receptor antibodies in the cerebrospinal fluid remained strongly positive. Subsequent ultrasound and MRI both suggested persistence of a left ovarian teratoma, prompting a second surgery 36 days later. During the second surgery, a 3 × 3 cm teratoma was found in the left ovary, and a left ovarian cystectomy was performed. The pathological findings were consistent with the previous result: a mature cystic teratoma without brain or neural tissue.

## 4 Discussion

Anti-NMDAR encephalitis is the most common autoimmune encephalitis (13), and it was first reported in 2007 (14). Advances in medicine have revealed that the presence of teratomas in female patients is a notable contributor to anti-NMDAR encephalitis. This teratoma-associated form of encephalitis presents with a broad spectrum of clinical manifestations, including psychiatric symptoms, seizures, cognitive decline, language disturbances, movement disorders, and other neurological deficits. If not diagnosed and treated promptly, the condition can lead to severe neurological sequelae or even prove fatal. Nevertheless, given the rarity of the disease, the existing literature is largely limited to individual case reports, which poses challenges in identifying common patterns across cases. Consequently, expanding the sample size to systematically investigate the shared characteristics of this condition is essential for deepening our understanding of the disease.

In our study, all 17 patients were young females with acute or subacute onset, most commonly acute. More than 70% presented with prodromal symptoms, including fever and/or headache. Among the 10 patients with headache, 7 had elevated CSF opening pressure, suggesting that headache may partly reflect intracranial hypertension in these patients. Psychiatric and behavioral abnormalities were the most common initial symptoms. The most prevalent clinical manifestations included psychiatric and behavioral disturbances, involuntary movements, seizures, and impaired consciousness (15–17). In addition to the presence of positive anti-NMDAR antibodies in the cerebrospinal fluid (CSF), our study also identified abnormalities, notably the presence of specific oligoclonal bands in the CSF of the majority of patients, which was consistent with previous research findings (5). However, due to the rarity of the condition, screening for anti-NMDAR antibodies in patients with ovarian teratoma is not indicated currently (18). Previous studies have shown that cranial magnetic resonance imaging (MRI) and electroencephalography (EEG) in patients with anti-NMDAR encephalitis often lack specific manifestations. In this study, cranial MRI revealed abnormal signals in more than half of the patients. EEG recordings from seven patients revealed diffuse or multifocal slow-wave activity, whereas the characteristic EEG patterns typically associated with anti-NMDAR encephalitis—such as extreme delta brush or delta waves—were observed in only four cases. Although these ancillary findings were generally non-specific, they still played an important role in differentiating this condition from other neurological disorders and provided valuable supportive evidence for the diagnosis. Despite updates to diagnostic and treatment guidelines for autoimmune encephalitis issued by obstetrics, gynecology, and neurology departments in 2022, the interval from symptom onset to diagnosis in teratoma-associated anti-NMDAR encephalitis often remains prolonged. This delay is largely attributable to the younger age at onset, the nonspecific nature of clinical manifestations, and the absence of distinctive findings on auxiliary examinations (10). In this study, the time from onset to diagnosis in 17 patients ranged from 5 to 34 days, with an average exceeding 2 weeks. Therefore, the possibility of this disease should be considered in patients suspected of having viral encephalitis or psychiatric disorders, especially young female patients. Clinicians should promptly test for anti-NMDAR antibodies in cerebrospinal fluid and conduct differential diagnosis as early as possible to avoid delayed management.

Approximately 80% of patients could recover if timely treatment is provided, and early diagnosis and intervention are important prognostic factors (10, 19). Current studies recommend transvaginal ultrasound as the first-line screening tool. When ultrasound fails to detect a suspected teratoma, more sensitive modalities such as CT or MRI should be considered (20, 21). In our study, Case 8 underwent three ultrasound examinations during hospitalization. The first two suggested a possible right ovarian cyst, while the third raised suspicion of a teratoma, which was later confirmed by MRI. Ultrasound findings are highly operator- and experience-dependent, suggesting that repeated imaging evaluations and a combination of modalities may enhance teratoma detection. Although pelvic MRI is generally considered superior to CT, pelvic CT remains a viable alternative in some cases due to its shorter scanning time and better tolerance in certain patients.

The primary treatments for teratoma-associated anti-NMDAR encephalitis include immunotherapy and tumor resection (22). Immunotherapy encompasses first-line therapy (corticosteroids, intravenous immunoglobulin, and plasma exchange), second-line therapy (rituximab, cyclophosphamide), long-term maintenance therapy (mycophenolate mofetil, azathioprine), escalation therapy (tocilizumab), and adjunctive therapy (methotrexate and bortezomib) (5, 10). These immunotherapies work by suppressing antibody production, thereby reducing the pathogenic impact of antibodies on neuronal receptors (22). All patients experiencing a first episode of anti-NMDAR encephalitis should receive first-line immunotherapy. If no improvement is observed after using two or more first-line therapies, second-line immunotherapy should be initiated promptly to improve patient outcomes (1). For patients who show little improvement after intensive first-line therapy or following second-line therapy, long-term maintenance immunotherapy may be considered. Escalation or adjunctive immunotherapy should be reserved for severe, refractory cases of encephalitis (10).

Early tumor resection has been shown to obviously improve outcomes in patients with teratoma-associated anti-NMDAR encephalitis (8, 23–25), whereas delayed resection is linked to a poorer prognosis (26). Therefore, it is widely accepted that systemic and neurological complications should not be regarded as absolute contraindications to surgery, even in patients with elevated anesthetic and surgical risks (27). All patients in our study underwent surgical intervention. Our findings underscore the need for a multidisciplinary approach involving neurology, intensive care, gynecology, and anesthesiology in the management of these patients. A comprehensive preoperative evaluation is crucial for developing a coordinated treatment strategy, establishing perioperative protocols, and preparing for potential emergencies. Surgery should be scheduled at an appropriate time after standardized systemic therapy. Intraoperatively, efforts should focus on minimizing operative duration while maintaining close monitoring of vital signs. In this study, two patients underwent surgery during the recovery phase, supporting the notion that surgical intervention at this stage is safe and feasible for those who respond well to immunotherapy (27).

Interestingly, in our study, two patients (Case 8 and Case 15) underwent two surgical procedures during their disease course. Case 8 initially underwent right salpingo-oophorectomy for a right ovarian teratoma; however, neither the disease course nor anti-NMDAR antibody showed improvement. Postoperative imaging revealed a left ovarian teratoma, prompting a second surgery for left-sided lesion resection. Case 15 required two left ovarian teratoma resections due to recurrent disease on the same side. In both cases, the lesions identified during the second operation were not detected during the initial surgical exploration. These findings suggest that although ipsilateral salpingo-oophorectomy may lower the risk of residual or recurrent microscopic disease on the same side, it does not eliminate the possibility of residual or newly developed lesions in the contralateral ovary. Therefore, during surgery, both ovaries should be systematically and thoroughly examined, with special attention paid to small or deeply embedded tumors. In cases where postoperative recovery is suboptimal, prompt pelvic imaging is recommended to rule out residual disease or newly developed lesions.

Additionally, this study identified two patients (Cases 5 and 6) with a prior history of teratoma who subsequently developed anti-NMDAR encephalitis triggered by a teratoma in the same ovary, indicating that a history of teratoma does not necessarily confer immunological safety. For patients wishing to preserve fertility, ultrasound surveillance every 6–12 months post-surgery is recommended. In contrast, for those without fertility intentions or with recurrent disease, ipsilateral salpingo-oophorectomy may be considered to lower the risk of encephalitis recurrence. Of note, both patients self-reported previous methotrexate treatment for ectopic pregnancy. However, detailed methotrexate administration timelines and cumulative doses were unavailable for two cases, as this information relied on patient self-reports and was documented only as part of their past medical history. Although the number of cases is small and lateral differences exist, the potential impact of ectopic pregnancy and methotrexate on the ovarian microenvironment and immune homeostasis warrants further investigation. Case 10 exhibited markedly elevated cerebrospinal fluid (CSF) pressure, reaching up to 357 mmH₂O, in the absence of prodromal symptoms such as fever or headache. This suggests that the lack of early warning signs does not necessarily correspond to a milder disease course. Furthermore, despite symptomatic improvement following treatment, the patient’s modified Rankin Scale (mRS) score remained at 5, indicating that elevated initial CSF pressure may serve as a potential predictor of poor prognosis in anti-NMDAR encephalitis.

Patients with teratoma-associated NMDAR encephalitis generally have a favorable prognosis (8). However, the mortality rate of severe anti-NMDAR encephalitis was about 6% (28). In the present study, only three patients showed good outcomes at 1 month postoperatively, and nearly half still exhibited severe neurological impairment at 3 months post-surgery. This indicates that although the long-term prognosis of teratoma-associated anti-NMDAR encephalitis is favorable, the functional recovery period is prolonged. Compared with previously reported cohorts (12, 24, 25), our patients demonstrated higher disease severity, as reflected by higher ICU admission (88% vs. 52.4–58.6%). The lower proportion of favorable outcomes at 3 months (24%) in oue study likely reflects the short follow-up duration and potential referral bias inherent to our tertiary center, rather than treatment failure. Recovery in anti-NMDAR encephalitis is known to be protracted, and longer follow-up is warranted (Table 4). Therefore, it is essential to fully communicate the expected rehabilitation timeline with patients’ families in clinical practice, adhere to long-term follow-up and rehabilitation interventions, and avoid premature discontinuation of treatment.

**Table 4:** Comparison of our cohort with published studies on ovarian teratoma-associated anti-NMDAR encephalitis.

Reference	Sample size	Age (Year)(median/mean)	Proportion with prodrome (fever/headache)	ICU admission rate (*n*, %)	Number of teratoma patients with surgery	Time to teratoma surgery (Day) (median/mean)	Extent of teratoma surgery	Follow-up time (Month)	Favorable outcome (*n*, %) (mRS ≤ 2)	Mortality	References
Dai 2019	29	23.14 ± 6.59	Fever: 22/29 (75.9%); Headache: 9/29 (31.0%)	17/29 (58.6%)	29/29 (100%)	–	–	37.69 ± 12.04	28/29 (96.6%)	1/29 (3.4%)	(12)
Jiang 2021	21	24.33 ± 5.12	13/21 (61.9%)	11/21 (52.4%)	20/21(95.2%)	36.92 ± 14.72(16–63)	6 patients: ovarian cystectomy; 14 patients: affected unilateral adnexectomy.	12	17/20 (85%)	0/20 (0%)	(24)
Iyengar 2022	23	26 (19–73)	–	–	23/23(100%)	–	8 patients: bilateral “full” oophorectomy or ovarian resection performed without pre-operative imaging evidence of teratoma.; 15 patients: Unilateral “partial” or “full” oophorectomies, and bilateral “partial” oophorectomies	12	14/23(61%)	–	(25)
Our study	17	25 (16–40)	12/17 (71%)	15/17 (88%)	17/17 (100%)	39 (17–143)	9 patients: Ovarian cystectomy; 8 patients: Salpingo-oophorectomy	3	4/17 (24%)	0/17(0%)	

This study has several limitations. First, because testing was performed by commercial laboratories, some results were qualitative, and cross-institutional titer comparisons were not feasible; Second, this was a retrospective record review with attendant missing data and a relatively short follow-up duration (3 months). Third, this was a single-center study with a small sample size, no control group, and potential referral bias from our tertiary neurological referral center, which may limit the generalizability of our findings to the broader population of teratoma-associated anti-NMDAR encephalitis. In addition, diagnostic tools and therapeutic protocols changed over the 10-year enrollment period. Furthermore, because headache features were not consistently documented in the retrospective records, we could not fully determine whether headache persisted into the encephalitic phase or its relationship with lumbar puncture timing and treatment response. Future multi-center prospective cohort studies with standardized long-term follow-up (≥24 months) and collaborative registry networks across tertiary and general gynecological centers are warranted to better characterize the recovery trajectory and enhance the generalizability of findings.

In summary, we first emphasized that timely pelvic imaging should be performed to rule out residual or newly developed lesions when postoperative recovery is unsatisfactory, as this may improve patient prognosis. Additionally, patients with a history of teratoma and prior methotrexate treatment may be at increased risk for teratoma-associated anti-NMDAR encephalitis. Although all patients showed improvement in at least one neurological symptom, only 24% achieved a favorable functional outcome (mRS ≤ 2) at 3 months, indicating a protracted recovery. Therefore, anti-NMDAR encephalitis associated with teratoma is characterized by acute onset and rapid progression, and clinical improvement can be achieved through multidisciplinary collaboration combined with prompt immunotherapy and tumor resection.

## Data Availability

The original contributions presented in the study are included in the article/Supplementary material, further inquiries can be directed to the corresponding author.
