# Supplementary material for: Clinical characteristics and treatment strategies of ovarian teratomas associated with anti-N-methyl-D-aspartate receptor encephalitis based on a case series
Source: Front Neurol. 2026 Oct 29;17:1817027. doi: 10.3389/fneur.2026.1817027 (PMC13754674; doi:10.3389/fneur.2026.1817027)
Supplement: Supplementary file 1 [file Table_1.DOCX]

Supplementary table 1 The change of pressure, WBC and protein in CSF

| Case No | Pressure (mmH₂O) | WBC (×10⁶/L) | Protein (mg/dL) |
| --- | --- | --- | --- |
| 1 | 330 | 90 | 31 |
| 2 | 135 | 32 | 19 |
| 3 | 210 | 89 | 89 |
| 4 | 155 | 145 | 54 |
| 5 | 130 | 16 | 51 |
| 6 | 240 | 16 | 32 |
| 7 | 150 | 6 | 16 |
| 8 | 170 | 69 | 40 |
| 9 | 210 | 36 | 23 |
| 10 | 357 | 40 | 21 |
| 11 | 200 | 20 | 18 |
| 12 | 150 | 12 | 20.4 |
| 13 | 250 | 19 | 29.5 |
| 14 | 200 | 64 | 34 |
| 15 | 320 | 22 | 39.6 |
| 16 | 185 | 31 | 19.8 |
| 17 | 145 | 57 | 60.5 |

Normal range: Pressure: 80-180mmH_2_O; white blood cell : 0-5×10^6^/L; Protein: 15-45mg/dL.

Supplementary table 2 The mRS for patients

| Case No. | Admission | After Medical Treatment | 1 Month after surgery | 3 Months after surgery |
| --- | --- | --- | --- | --- |
| 1 | 5 | 5 | 5 | 5 |
| 2 | 5 | 5 | 5 | 5 |
| 3 | 5 | 1 | 0 | 0 |
| 4 | 5 | 5 | 5 | 5 |
| 5 | 3 | 1 | 0 | 0 |
| 6 | 5 | 5 | 4 | 3 |
| 7 | 5 | 5 | 1 | 0 |
| 8 | 5 | 5 | 5 | 5 |
| 9 | 5 | 5 | 4 | 3 |
| 10 | 5 | 5 | 5 | 5 |
| 11 | 5 | 5 | 4 | 3 |
| 12 | 5 | 5 | 4 | 1 |
| 13 | 5 | 5 | 4 | 4 |
| 14 | 5 | 5 | 5 | 5 |
| 15 | 5 | 5 | 5 | 5 |
| 16 | 5 | 5 | 5 | 5 |
| 17 | 5 | 5 | 5 | 4 |
